# Plasmonic Nanostructures for Nano-Scale Bio-Sensing

**DOI:** 10.3390/s111110907

**Published:** 2011-11-21

**Authors:** Taerin Chung, Seung-Yeol Lee, Eui Young Song, Honggu Chun, Byoungho Lee

**Affiliations:** 1 National Creative Research Center for Active Plasmonics Application Systems, Inter-University Semiconductor Research Center and School of Electrical Engineering, Seoul National University, Gwanak-Gu Gwanakro 1, Seoul 151-744, Korea; E-Mails: taerinc@gmail.com (T.C.); ssodra87@snu.ac.kr (S.-Y.L.); song9758@snu.ac.kr (E.Y.S.); 2 Department of Biomedical Engineering, Korea University, Seoul 136-701, Korea; E-Mail: bioneer@gmail.com

**Keywords:** surface plasmon resonance, optical sensors, plasmonic sensors, nanostructure

## Abstract

The optical properties of various nanostructures have been widely adopted for biological detection, from DNA sequencing to nano-scale single molecule biological function measurements. In particular, by employing localized surface plasmon resonance (LSPR), we can expect distinguished sensing performance with high sensitivity and resolution. This indicates that nano-scale detections can be realized by using the shift of resonance wavelength of LSPR in response to the refractive index change. In this paper, we overview various plasmonic nanostructures as potential sensing components. The qualitative descriptions of plasmonic nanostructures are supported by the physical phenomena such as plasmonic hybridization and Fano resonance. We present guidelines for designing specific nanostructures with regard to wavelength range and target sensing materials.

## 1. Introduction

As nano-fabrication processes have rapidly developed, the recent sensor technologies are being used for reading DNA bases [1] as well as detecting protein-protein interactions [2,3], surface membrane binding events [4] and antigen-antibody recognition events [5]. In particular, the demand for surface plasmon resonance (SPR)-based nano-scale bio-sensing has increased due to the advantage of label-free, minimal interference, and real-time monitoring performance [6]. The conventional SPR sensor originates from propagating surface plasmons. This plasmon can be described as surface plasmon polaritons in optically thin metal film, usually noble metal layers. Propagating plasmon waves can be produced in various illumination configurations from grating coupling to near-field excitation. The universal scheme for SPR sensing is the Kretschmann geometry where a thin noble metal film is covered on a prism. However, it has drawbacks in applications due to its bulky system and low spectral resolution [7,8]. On the other hand, localized SPR (LSPR), a coupling between electromagnetic field and spatially confined free-electrons, has a potential for resolving these issues in an attempt to detect nano-scale biological interactions. LSPR sensing structures are typically fabricated on a chip where noble metal nanostructures are coated or patterned on a dielectric substrate. It seems feasible that sensor system can be miniaturized by nano-scaled localized plasmons installed on effectual microspectroscopy. Since the resonance condition of LSPR is determined by the electron motions, optical properties of this sensing scheme are highly dependent on the geometry of metallic nanostructures. Such nanostructures for achieving LSPR can resonate with the incidence of electromagnetic fields at certain wavelengths, giving rise to strongly enhanced near-fields [9,10]. Plasmon excitations on the metallic nanostructures can be a promising constituent of the propagating plasmon employed in traditional SPR sensors. As compared to SPR sensors, LSPR sensors can be advantageous due to their capability of optimizing the sensing performance through variations of the size and shapes of nanostructures. The extremely intense and highly confined electromagnetic fields induced by the LSPR can realize a highly sensitive probe to detect small changes in the dielectric environment around the nanostructures. When the biomolecular binding events get close to the surface of a noble metal nanostructure, the refractive index of immediate environment surrounding the nanostructure is increased. Thus, biomolecular interactions at the surface of the nanostructures directly lead to local refractive index changes; these changes can then be monitored via the LSPR peak wavelength shift. This can allow for the detection of extremely low concentrations of molecules with surface-enhanced Raman scattering (SERS) [11,12] as well. Hence, the ideal LSPR nanosensor should have a high spectral shift along the alteration of surrounding material and a narrow linewidth of spectral response [13]. Yet, lower sensitivity has been marked at LSPR sensors compared with their counterparts.

Major issues of current LSPR bio-sensor research include understanding LSPR properties in certain nanostructures, optimizing the design of nanostructures, and improving sensitivity and detection limits. In this review, the uses of assorted nanostructures as potential sensing components are presented and re-categorized according to their similar characteristics. Exemplary cases of biological sensing with LSPR are addressed.

## 2. Basic Principle of Localized Surface Plasmon Resonance

When a metallic nanostructure is illuminated by an appropriate incident wavelength, localized electrons in the metallic nanostructure oscillate and create strong surface waves [14]. The curved surface of the particle generates an effective restoring force on the conduction electrons so that resonance can arise. This phenomenon leads to strong field enhancement in the near field zone. This resonance is called LSPR. The LSPR phenomenon is theoretically possible in any kind of metal, semiconductor or alloy with a large negative real part and small imaginary part of electric permittivity.

We can obtain the explicit form of electromagnetic field distribution using some assumptions when a particle interacts with electromagnetic field. First, we assume the particle size is much smaller than wavelength of light in the surrounding medium. In this condition, the phase of the harmonically oscillating electromagnetic field is approximately constant over the particle volume. This is called quasi-static approximation. Second, we choose a simple geometry for analytical treatment: The particle is a homogeneous isotropic sphere of radius *r*_0_, and surrounding material is a homogeneous, isotropic and non-absorbing medium. On the illumination of static electric fields, we solve Laplace equation for the potential, ∇^2^*V*=0. Due to the azimuthal symmetry of the problem and requirement that the potentials remain finite at the center of the particle, the solutions of this Laplace equation for potentials inside and outside the particle can be written as:

(1) $$\begin{array}{l} V_{\text{in}}(r,\theta )=\sum_{l=0}^{\infty }A_{l}r^{l}P_{l}(\text{cos}\;\theta ),\\ V_{\text{out}}(r,\theta )=\sum_{l=0}^{\infty }\left[B_{l}r^{l}+C_{l}r^{-(l+1)}\right]P_{l}(\text{cos}\;\theta ). \end{array}$$

where, P*_l_*(cos*θ*) is the Legendre polynomial of order *l*, and *θ* is the angle between the position vector *r* and the electric field **E⃗**. The coefficients *A_l_*, *B_l_*, *C_l_* can be determined using boundary conditions: as *r* approaches infinity, the potential approaches –| **E⃗** | *r*cos*θ*. The tangential components of electric fields are equal at *r = r*_0_, and normal components of electric flux density are equal at *r = r*_0_. The solutions are:

(2) $$\begin{array}{l} V_{\text{in}}=-\frac{3\varepsilon_{d}}{\varepsilon_{m}(\omega )+2\varepsilon_{d}}\left|\vec{E}\right|r(\text{cos}\;\theta ),\\ V_{\text{in}}=-\left|\vec{E}\right|r\;\text{cos}\;\theta +\frac{\varepsilon_{m}(\omega )-\varepsilon_{d}}{\varepsilon_{m}(\omega )+2\varepsilon_{d}}\left|\vec{E}\right|r_{0}^{3}\frac{\text{cos}\;\theta }{r^{2}}. \end{array}$$

Here, *ε*_m_(*ω*) and *ε*_d_ are electric permittivity of metal and surrounding dielectric layer, respectively.

We can interpret *V*_out_ physically: *V*_out_ is the superposition of the applied field and a dipole induced by this field. Therefore, we introduce the dipole moment **p⃗** as:

(3) $$\vec{p}=4\pi \varepsilon_{0}\varepsilon_{d}r_{0}^{3}\frac{\varepsilon_{m}(\omega )-\varepsilon_{d}}{\varepsilon_{m}(\omega )+2\varepsilon_{d}}\vec{E}.$$

If we introduce polarizability *α* via **p⃗** = ε_0_ε_d_*α***E⃗**, we can express *α* as:

(4) $$\alpha =4\pi r_{0}^{3}\frac{\varepsilon_{m}(\omega )-\varepsilon_{d}}{\varepsilon_{m}(\omega )+2\varepsilon_{d}}.$$

We can expand Equation (4) in case of arbitrary shaped particle as [10]:

(5) $$\alpha =(1+\kappa )\varepsilon \Omega \frac{\varepsilon_{m}(\omega )-\varepsilon_{d}}{\varepsilon_{m}(\omega )+\kappa \varepsilon_{d}}.$$

where Ω is the volume of the particle. As can be seen, the dipolar polarizability *α* could be maximized at the condition of Re(*ε*_m_(*ω*)) = −*κε*_d_, which is denoted by the resonance condition of LSPR assuming Im(*ε*_m_(*ω*)) is relatively small and constant value with the variation of frequency. *κ* is a shape factor that embodies geometrical polarizability of the surface that indicates the electron oscillations. The shape factor of a small nanostructure plays a critical role to increase dipolar polarizability for enhancing LSPR strength. This variable can be straightforwardly expressed by aspect ratio [15]. In other words, resonant enhancement increases as particles are made more needle-like. A nanorod is designed for achieving higher aspect ratio in that dipolar polarizability linearly depends on the aspect ratio of a nanostructure. The notable increase of aspect ratio can lead to the sensitivity improvement accompanied by remarkable wavelength shift [16].

## 3. Design Principles for LSPR Sensing

In this section, we will introduce the various principles for designing the highly sensitive and strongly enhanced LSPR sensors. In attempt to compare sensing performance, several major factors such as sensitivity, figure of merit (FOM) and resolution will be introduced.

First of all, sensitivity *S* is defined as the ratio of resonant wavelength shift ∂*λ_res_* to the variation of surrounding refractive index ∂*n_s_*:

(6) $$S=\frac{\partial \lambda_{\mathrm{res}}(nm)}{\partial n_{s}(\mathrm{RIU})},$$

where RIU means refractive index unit. FOM is considered as a useful parameter in verifying LSPR nanosensor. It is defined as the ratio of the refractive index sensitivity to the resonance width Δλ:

(7) $$\mathrm{FOM}=\frac{S\left(\mathrm{nm}\cdot {\mathrm{RIU}}^{-1}\right)}{\Delta \lambda (nm)}.$$

Resolution is typically defined as the minimum detection limit. Unlike the conventional propagating SPP-based sensors, localized SPPs have their own resolution for detecting the signals. This can be improved by the size and geometry of nanostructure. These factors can describe the sensing performance and be used in evaluating various types of sensors. Many groups have exploited various nanostructures in attempt to improve LSPR sensor with regard to high sensitivity, FOM and spectral resolution.

### 3.1. The Use of Inter-Coupling between Nanoparticles

**Figure 1.**
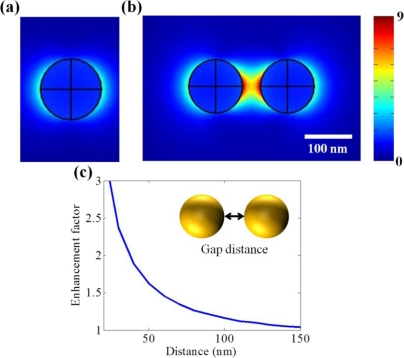
Electric field distributions (V/m) at **(a)** a single Au nanoparticle and **(b)** nanoparticle dimer when the incident wavelength of light is 633 nm and the magnitude of incident electric field is 1 V/m. The diameter of Au nanoparticle is 100 nm. These calculations are based on finite element method; **(c)** Field enhancement factor of nanoparticle dimer with respect to the single nanoparticle. In all simulations in this figure, the radius of each nanoparticle is 50 nm.

One of the earliest LSPR sensor nanostructures was the simple sphere-shaped nanoparticle which has the shape factor of 2. Figure 1(a) presents the localized surface plasmon field induced by a single nanoparticle. However, it shows that the field strength is too weak and field distribution is not confined to certain point so that it does not seem to be appropriate for detecting small volumes of bio-molecules.

Otherwise, additional field enhancement can be achieved by inter-coupling between particles when nanoparticles are close to each other. From the inter-coupling between two nanoparticles, stronger LSPR enhancement can be achieved based on the localized capacitive coupling at the nano gap surrounded by nanoparticles as shown in Figure 1(b). Such LSPR enhancement originates from the charge induction between two nanoparticles, which interact stronger as they get closer to each other. This relation between the field enhancement factor of nanoparticle dimer and gap distance is clearly shown in Figure 1(c). A metallic nanoparticle array has been proposed extending from this phenomenon. LSPR sensing behaviors can be verified investigating on the resonant spectrum of metallic nanoparticle array. The number and position of nanoparticle array is arbitrarily varied in attempt to identify the optical resonant response.

**Figure 2.**
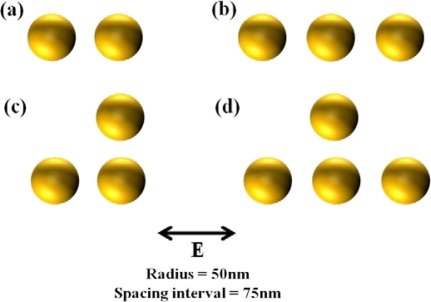

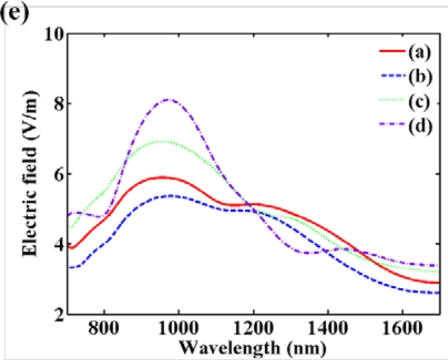
Array type of nanoparticles: **(a)** two nanoparticles; **(b)** three nanoparticles arrayed in a transverse direction; **(c)** three nanoparticles arrayed in the shape of a right triangle; **(d)** four nanoparticles arrayed in the shape of an isosceles triangle; **(e)** LSPR resonant spectra with respect to the number and position of nanoparticles. Incident electric field is 1 V/m.

Figure 2 provides the resonant spectra of various types of nanoparticle arrays. Although LSPR resonant wavelength is not so much shifted with the variation of the number of nanoparticles, it can be shown that the peak of the resonance gets sharper due to the increase of particle interactions.

It is also important that the shift of the resonant wavelength is negligible when the spacing interval is higher than the diameter of nanoparticles. This uniformity characteristic is required for nanoparticle array as a bulk refractive index sensor [17–19]. A bulk refractive index sensor has been typically characterized by detecting the difference of surrounding refractive index.

### 3.2. Structural Modification for Higher Sensitivity

#### 3.2.1. Increase of Polarizability via Multipolar Resonance

**Figure 3.**
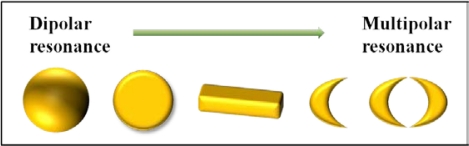
Schematic diagram for describing the polar properties of various nanostructures that are used in LSPR sensors.

In a notable way to realize more advanced LSPR sensors, one of the useful methods is increasing the polarizability by employing the multipolar resonance as depicted in Figure 3. Fundamentally, a nanosphere exhibits dipolar resonance with degenerated longitudinal and transverse modes due to the spherical symmetry. However, by the increase of aspect ratio at a nanoparticle, both transverse and longitudinal modes are split. From these two modes, two plasmon resonant peaks are observed at nanorods due to anisotropy [20]. Each resonant peak in two plasmon modes corresponds to longitudinal and transverse plasmon modes, respectively. Derived from this concept, multipolar resonances are introduced in diverse nanostructures [21]. A nanodisk displays the anisotropic property distinct from a nanoparticle.

**Figure 4.**
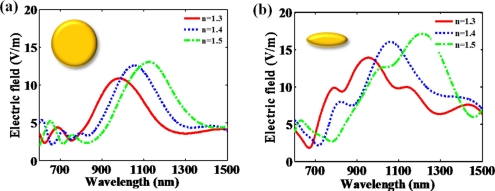
Optical spectra in response to the variation of surrounding refractive indexes from 1.3 to 1.5 at the nanodisk structure with respect to **(a)** aspect ratio 1:1 and **(b)** aspect ratio 5:1. Incident electric field is 1 V/m.

Hanarp *et al*. reported that the optical extinction peak as a function of refractive index is more shifted as the aspect ratio of a nanodisk increases [22]. Figure 4 presents that optical spectrum differently responds with regard to the aspect ratio of the nanodisk. The elongated nanodisk made by the increase of aspect ratio shows that the resonant peak is more shifted than the resonant peak of circular nanodisk. It can be explained that higher aspect ratio makes stronger dipole moment and acutely influences on the surrounding materials. It makes the resonance more sensitive to the refractive index changes. In addition, the magnitude of induced fields is increased due to stronger dipole moment.

**Figure 5.**
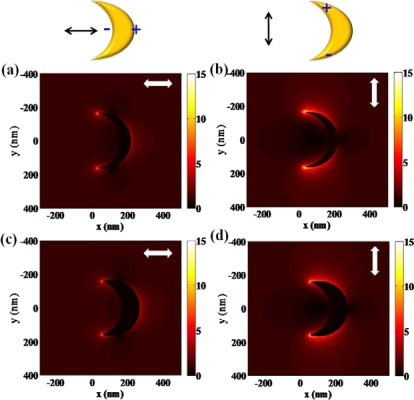
Intensity distributions (arbitrary unit) of nanocrescent with respect to the width of nanocrescent and incident polarization state of light. **(a)** 50 nm wide and horizontally polarized light; **(b)** 50 nm wide and vertically polarized light; **(c)** 100 nm wide and horizontally polarized light; **(d)** 100 nm wide and vertically polarized light.

High refractive index sensitivity and corresponding FOM are attributed to plasmonic nanostructures associated with multipolar resonances. One of the representative structures which enhance its sensitivity by increasing the polarizability is the nanocresent structure [3,23]. Adopting a sub-10 nm sharp edge or tip, extremely high field intensity can be achieved at the nanocrescent. Localized and confined electronmagnetic field enhancement, called “hot spot”, is attracting much attention in designing sensors for bio-molecule detection as well. Hot spots generated at nano-scale sharp tips can provide localized molecular sensing with the greatest sensitivity and resolution. As shown in Figure 5, localized field intensity is much larger at the edges of a nanocrescent. The shape and dimensions of a nanocrescent should be precisely designed to achieve localized field enhancement at the sharp edges. One of the major physical parameters for designing a nanocrescent is the width of nanocrescent since it strongly relates to the structural polarizability. Figure 5(a,b) show the field distribution near the nanocrescent with the width of 50 nm, whereas Figure 5(c,d) show that of 100 nm. By comparing the two structures, it can be shown that the local field intensity is slightly larger at the 50 nm width nanocrescent. This can be explained by the relation between shape factor and aspect ratio, which means that more needle-like structure can strongly enhance the field. In addition, field enhancement of nanocrescent is strongly dependent on the polarization state of light. When illuminated by vertically polarized light, higher field intensity can be achieved as compared with the incidence of horizontally polarized light. This phenomenon can result from the difference of accumulated electrons at the sharp edges of a nanocrescent. Horizontally polarized light equally separates the electrons to the both ends of nanocrescent, whereas vertically polarized light enables electrons to totally move to sharp edges of a nanocrescent, which leads to higher field intensity.

Moreover, some structures can be explained by the composition of both multipolar resonance and inter-coupling of nanoparticles. For example, double nanocrescents structure facing each other is a representative case.

**Figure 6.**
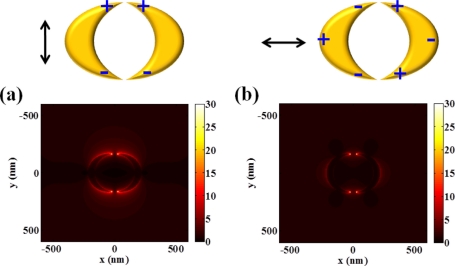
Intensity distributions (arbitrary unit) of double-crescents with respect to incident polarization state of light: **(a)** vertically polarized light and **(b)** horizontally polarized light. The width of a nanocrescent is fixed to 50 nm.

As described in Figure 6, intensity distributions of double crescents are similar to the optical responses of a single nanocrescent and sensitively respond to incident polarization of light. However, they can produce much higher field intensity.

Enhanced field intensity calculated at double nanocrescents is approximately two times larger than that of single nanocrescent for the vertically polarized case, and even higher for the horizontally polarized case. It can support that localized capacitive inter-coupling nestled at nanogaps enhances even more the field intensity of a single nanocrescent structure. This inter-coupling effect is more effectively generated with horizontally polarized light, which can be explained by the attraction between the oppositely induced charges at the end of nanocrescents.

#### 3.2.2. The Use of Plasmon Hybridization for Enhancing LSPR Resonances

**Figure 7.**
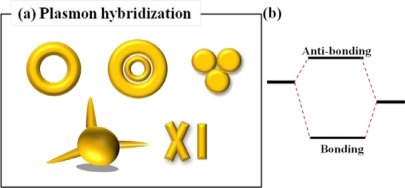
**(a)** Various nanostructure designs based on the plasmon hybridization and **(b)** simplified energy diagram for explaining the plasmon hybridization.

Another promising method for increasing the sensitivity of LSPR sensor is using the plasmon hybridization [24]. Schematic diagram of representative nanostructures for LSPR sensing which is based on plasmon hybridization is shown in Figure 7(a). Plasmon hybridization theory originates from the hybridization of essentially fixed-frequency plasmon resonances of individual nanostructures in a complicated nanostructure. In the model of plasmon hybridization, bonding and anti-bonding modes are introduced. At the low energy level, bonding mode is formed by symmetrical coupling in a nanostructure. Meanwhile, anti-bonding mode results from asymmetrical coupling in a nanostructure at high energy level. The highly geometry-dependent plasmon response can be seen as an interaction between two conjugated physical modes as mentioned above. The physical models of plasmonic hybridization have been incessantly brought into novel nanostructures such as nanorings and nanoshells. This model can be employed to take account of the sensitive geometrical tunability of the plasmon resonant frequency of metal-based complex nanostructures [25]. The plasmon hybridization picture in Figure 7(b) can be used to identify the multi-featured plasmon responses of more complex metallic nanostructures, such as nanoring, trimers and other nanoparticle aggregates. Intentionally, dual or multiple resonant wavelength sensing can be achieved by nanodisk trimers [26], nanoring trimers [27], and plasmonic oligomers [28]. Interaction of incident light with gold nanodisk trimers can give rise to dual wavelength sensing. As the inter-gap distance among nanodisk trimers increases, dual resonant peaks in visible and near infrared wavelength range are observed arising from the electromagnetic coupling among the three disks.

**Figure 8.**
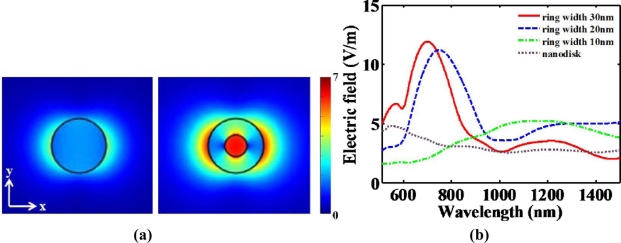
**(a)** Induced electric field distributions (V/m) of nanodisk and nanoring structure, respectively, at *z* = 25 nm. The outer radius and thickness of nanodisk and nanoring are fixed at 50 nm, respectively. The width of nanoring is fixed at 30 nm. Incident electric field is 1 (V/m). Surrounding medium is considered as an air (*n* = 1); **(b)** Spectrum of enhanced amplitude with respect to the variation of inner radius at nanoring.

Moreover, Tao *et al*. reported that the sensitivity of nanoring trimers to the refractive index of the surrounding medium is nearly three times larger than that of nanodisk trimers of comparable size [27]. This results from the plasmonic hybridization with a great tunability (ring size, wall thinkness and ring separation). Due to complementary vibrational analysis of biomolecules combined with LSPR, the enhancement of scattering light from such trimers can play a crucial role in the field of biological sensing. Hence, multiple resonant wavelength sensing has originally developed from the complexed nanostructures. This sensing method has been more distinguished for multiplexed bioassay. Tunable plasmonic nanostructures consisting of periodic arrays of nanodisks and nanorings have also been investigated theoretically and experimentally [2,29]. A nanoring, which is more complicated than a nanodisk, can be fabricated by colloidal lithography and ion beam etching. When it is necessary to have nano-scale small volumes for the detection of unlabeled, binding biomolecules, a nanoring may confine the detection volume and be plugged in quantified biosensing applications. As shown in Figure 8(a), a nanoring can confine induced electric fields into the nano-scale area as compared to a nanodisk. Additional field enhancement can be achieved at the center of a nanoring due to the plamonic hybridization. In designing the nanoring structure, nanoring width must be the critical parameter. Figure 8(b) describes that narrower spectral linewidth at the visible-NIR spectrum can be obtained as the nanoring width increases. Narrow spectral linewidth can be mentioned as high spectral resolution. Spectral resolution at the nanoring might be determined by the nanoring width. It also implies that this structure can have more degree of freedom than the nanodisk. However, the fabrication of nanoring structure should be deliberately processed to make sure of a certain nanoring width.

Other representative nanostructures based on plasmonic hybridization are the nano-star [30] and nano-pillar [31] structures. Hao *et al*. addressed the nanostar structure explicitly separating local excitations [30]. A nanostar is composed of a central core from which a number of protruding tips extend. They typically show an LSPR of the core and multiple LSPRs corresponding to the tips and core-tip interactions. The latter are polarization dependent and accompanied by large local electric field enhancements at the sharp ends of the tips. They revealed that several different plasmon modes can be experimentally observed for a specific polarization state of the incident light. It is described by the hybridization of plasmons associated with the core and individual tips of the nanoparticle. The electric near-field enhancement arisen from tip plasmon modes is determined by the size of the core, as a result of the hybridization of tip and core modes.

**Figure 9.**
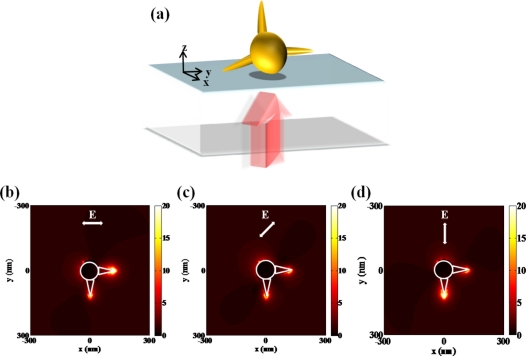
**(a)** Schematic of simplified nanostar placed onto a glass substrate. Electric field distributions at a distance of 10 nm from a nanostar when illuminated by **(b)** horizontally polarized light; **(c)** 45° polarized light; and **(d)** vertically polarized light.

Individual plasmonic hot spots in a single gold nanostar can be selectively produced at the tips with respect to the wavelength and polarization of incident light [32]. In order to investigate the optical behavior of a nanostar in visible range, electric field distributions from a simplified nanostar structure are numerically calculated in Figure 9. Unique property of three-dimensional structure can generate specific field pattern with the variation of the polarization state. Resonant frequency or wavelength is based on the length scale of individual tips assuming that core dimension is fixed. Due to the complicated nano structures such as nanostar, a couple of plasmonic modes are generated. Each plasmonic mode is closely correlated to the excited state of unit structure, corresponding to incident polarization direction of light.

### 3.3. Sensitivity Improvement Utilizing Fano Resonance and Electromagnetically Induced Transparency

Many researchers have paid attention to plasmonic structures that exhibit Fano resonances in their optical spectra as a sensing platform. Fano resonance emerges from the coherent coupling and interference of bright and dark plasmon modes [33]. A weak coupling and interference between a dark and bright plasmon modes is fundamentally required for a Fano resonance. This coupling results from symmetry breaking [34]. Another approach which can introduce a coupling between dark and bright modes is launching an anisotropic environment, such as depositing a nanoparticle, or fabricating a nanostructure by deposition methods, onto a dielectric substrate. This is mentioned as substrate-induced Fano resonance as well. For a spherical nanoparticle in a vacuum, there is no coupling between the bright dipolar and dark quadrupolar modes. However, when a nanoparticle is placed onto the dielectric substrate, the image of a dipolar plasmon will obtain an established quadrupolar field component across the nanoparticle, introducing a coupling between its dipolar and quadrupolar plasmons. The resulting plasmon modes will be the superposition of both dipolar and quadrupolar modes. They have appeared to be surprisingly more sensitive to the local dielectric environment than the instinct plasmon modes of the nanostructure. In addition, electromagnetically induced transparency (EIT) phenomenon can be manipulated by symmetry breaking. It is known as the elimination of absorption via quantum interference in an atomic medium. Narrow transparency resonance in the absorption spectrum is characterized by this phenomenon. This phenomenon would be desirable for sensing applications due to narrow spectral resonant width. Liu *et al*. demonstrate that a plasmonic EIT analogue can be achieved using a planar complementary metamaterial which consists of cut-out structures in a homogeneous gold film [35].

### 3.4. Experimental Measurements of Various Nanostructures as LSPR Nanosensors

**Figure 10.**
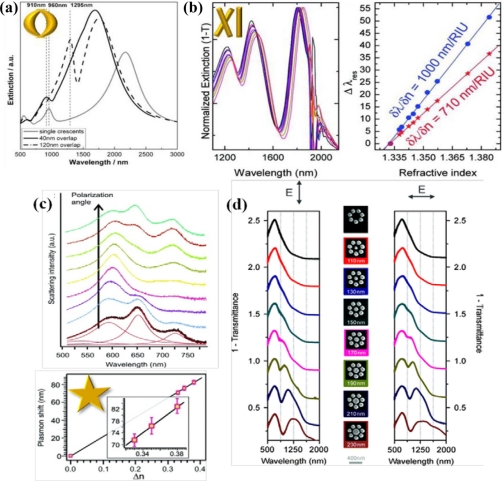

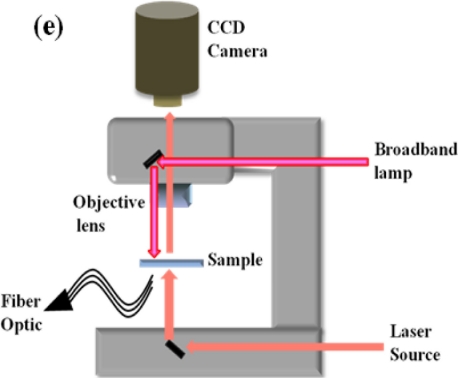
Various nanostructures as the candidate of LSPR nano biosensor. **(a)** Ultraviolet-visible-near-IR extinction spectra of stacked double crescents; **(b)** extinction spectra and refractive index sensing results of XI cavity; **(c)** light scattering spectra of a single gold nanostar and average spectral shift of nanostars resonances as a function of surrounding refractive index; **(d)** extinction spectra of plasmonic octamers depending on the center particle diameter for vertical and horizontal polarizations and **(e)** schematic of the measurement system for LSPR sensing. Figures are reprinted with permission from [28,36–38]. (a) Copyright (2011) American Chemical Society (b) Copyright (2011) American Chemical Society (c) Copyright (2010) American Chemical Society (d) Copyright (2011) American Chemical Society.

A variety of nanostructures have been experimentally exploited for the potential performance of LSPR nanosensors recognizing the variation of local refractive index. The aforementioned nanostructures, double nanocrescents and nanostar, are experimentally demonstrated [36,37]. Derived from single nanocrescent, stacked double nanocrescents were implemented by colloidal lithography. The close proximity of the nanocrescents leads to a new resonant coupling process. Additional multiple plasmonic resonances are brought into double crescents. The plasmon hybridization picture can also be applied to provide the physical description of double nanocrescents. Figure 10(a) shows that the overlapping of double nanocrescents can create a dual resonant wavelength mode. The overlapping difference causes a change in shift direction with respect to a single crescent resonance. The hybridization of nanocross and nanobar (XI) can take to the coherent coupling of both bright and dark plasmon modes [38]. This proposed structure might provide voluminous sensing areas by introducing a selective dry plasma etching of the substrate which can be applied to a wide range of LSPR as well. When placed on a dielectric substrate, the image of dipolar plasmon will bring into a significant quadrupolar field component across a nanostructure, launching a coupling between dipolar and quadrupolar plasmons. The physical explanation of this structure can also be supported by Fano resonance. Figure 10(b) shows the experimental results for the 20 nm gap XI array. Three hybridized modes, characterized by the nanocross and nanobar, are observed at extinction spectra. Multiwavelength spectroscopic sensing would be obtainable. The detailed comparison of the bulk LSPR refractive index sensitivities was conducted. Different glycerol solutions from 0% to 40% were supplied by a microfluidic chip. Triple hybridized modes lead to achievement of high sensitivity up to 1,000 nm/RIU with this structure. Multiple resonance sensing can be observed at the nanostars provided in Figure 10(c). Multiple LSPRs with respect to the tips and core-tip interactions are monitored from scattering spectra. The longer wavelength resonances are polarization dependent. The tips or the interaction between tips and core strongly influence on the longer wavelength resonances. Each plasmonic mode is closely correlated to the excited state of unit structure, with respect to incident polarization direction of light. Refractive index sensing was conducted by glucose water solutions with refractive indexes ranging from 1 to 1.38. One of remarkable nanoparticle aggregates is plasmonic oligomers, representing tailored optical properties [28]. Figure 10(d) shows the role of a center nanoparticle in determining the resonant behavior of plasmonic oligomers. Fano resonance becomes more dominant as the center particle diameter gets enlarged over 150 nm. A variety of array structures have been experimentally verified as well. Periodic gold nanopillar system has been reported to introduce complementary properties of localized and extended surface plasmons together [39]. This system is characterized by monopole antenna arrays. Aligned gold nanotube arrays have been presented as competitive refractive index sensors [40]. It claims that as the nanotube walls are exposed, the sensing characteristic of inside and outside walls is differentiated.

Kabashin *et al*. introduced plasmonic nanorod metamaterials for biosensing [41]. They demonstrated sensing improvement utilizing a plasmonic metamaterial that is subject to supporting an anisotropic guided mode in a porous nanorod layer. By overlapping between the probing field and the active biological substance, enhancement of sensitivity can be achieved after providing a strong plasmon-mediated energy confinement. A typical measurement system for LSPR refractive index sensing is based on a standard upright microscopy as shown in Figure 10(e). This setup can provide spectral measurements from a broadband lamp and bright-field image capture by the use of CCD camera. These notable nanostructures such as double nanocrescent, nanostar and plamsonic oligomers are especially characterized by multiple resonant wavelengths. Multiple resonant modes can bring into large spectral shift caused by the refractive index change.

## 4. Nano-Scale Applications of LSPR Sensors

### 4.1. Optical Antenna for Single Molecule Detection

**Figure 11.**
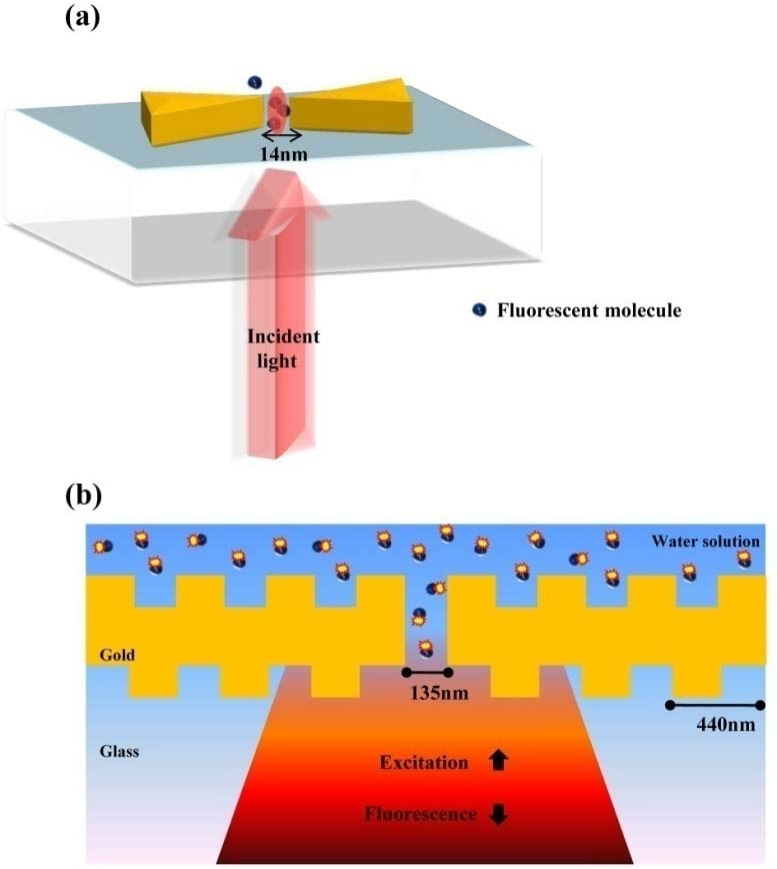
Single molecule detection: **(a)** single-molecule fluorescence enhancements produced by a bowtie nanoantenna; **(b)** unidirectional fluorescence emission of molecules in a nanoaperture with plasmonic corrugations.

Optical antennas analogous to microwave antennas have been viewed as the representative nanostructure for single molecule detection. Optical fields can be confined to subdiffraction-limited volumes and can be enhanced by the use of optical antennas [42–44]. The unique characteristics of optical antennas allow detecting a few nano-scale molecule units. It is well known that the field excitation and angular directivity of a nano-antenna can be controlled by the physical variables of the antenna structure in analogy to microwave antennas. One of best nanostructures as an optical antenna is a bowtie-shaped nanostructure [45]. Bowtie nanostructures are characterized by symmetrical sharp tips, as compared to nanorods referred to as optical dipole antenna. Kinkhabwala *et al.* reported that gold bowtie nano antenna shows the enhancement of single molecule's fluorescence up to a factor of 1,340, ten times higher than previously experimented [46]. A single fluorescent molecule with transition dipole behaves as a nanoscale optical sensor of the local electric field near a bowtie nanoantenna as presented in Figure 11(a). Figure 11(b) presents a configuration for enhancing and controlling the fluorescence emission of molecules diffusing in the central aperture reported by Aouani *et al* [47]. Fluorescent molecules are coupled to a single nanoaperture surrounded by periodic corrugations etched in a thick gold film. The periodic circular corrugations act as a grating antenna reversibly coupling light to surface plasmon wave.

From this grating antenna, much higher transmission can be obtained through these corrugated apertures than with standard noncorrugated apertures. In addition, they can achieve bright emission and narrow directionality, which contribute to improving the detection of single molecules. Thus, corrugated nanoapertures can provide a source for intense fluorescence light with narrow directionality.

**Figure 12.**
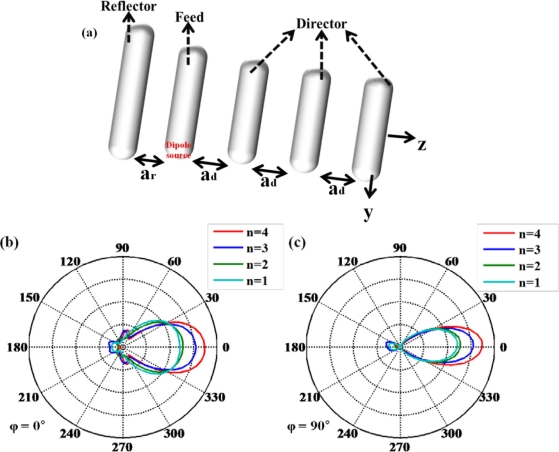
**(a)** Schematic of optical Yagi-Uda antenna. Feed length of optical Yagi-Uda antenna, wavelength of source, parameters *a_r_*, and *a_d_* are set to 160 nm, 570 nm, 130 nm, and 143 nm, respectively. Polar radiation patterns with respect to the number *n* of directors **(b)** at ϕ = 0° (*y*-*z* plane) and **(c)** ϕ = 90° (*x*-*z* plane).

The optical Yagi-Uda antenna has been described as a fascinating nano-antenna, just like the radio frequency (RF) Yagi-Uda antenna [48,49]. This nano-antenna can be applied to the technique of single molecule detection due to directional far-field radiation. It is composed of an actively driven feed element surrounded by a set of parasitic elements performing as reflectors and directors as shown in Figure 12(a). Directional radiation from an optical Yagi-Uda antenna can be manipulated by the number of directors and the length of reflector. Directors help emitting light from feed element to propagate far more. Figure 12(b,c) indicate the angular directivity of an optical Yagi-Uda antenna with the variation of the number of directors. It can be shown that the main lobe of the radiation pattern becomes narrower and longer as the number of directors increases. Other important design parameters for a Yagi-Uda antenna such as the lengths of the reflector and director are set to 1.05× (feed length) and 0.9× (feed length), respectively. These values are chosen from [49], where they are optimized by the design rules used in the microwave region.

### 4.2. Biological Applications with LSPR Nanosensors

The differences of local refractive index following biological interactions can be discerned utilizing the exclusive properties of LSPR. Qualitative detection of biological interactions can be characterized by employing organic molecules. Most organic molecules have a higher refractive index than buffer solution. When organic molecules bind to nanoparticles, the local refractive index increases accompanied with a red shift of the extinction spectrum. This mechanism makes possible the recognition of biomolecules. Biomolecular recognitions have become imperative in clinical diagnostics as well as in pharmacology [50,51]. In particular, LSPR acutely responds to the reactions occurring at the surface of metallic nanostructures. LSPR nano-sensors can detect lipid membranes, protein binding and up to DNA. A ligand-receptor interaction which appears on the plasma membrane can also be probed with LSPR [4]. In an attempt to achieve fast, quantitative screening of ligand-receptor interactions, lipid bilayers that resemble the native membrane environment must be probed by large arrays of nanostructures illuminated by light. Thus, LSPR sensing technology is necessary to analyze membrane proteins in native environments.

**Figure 13.**
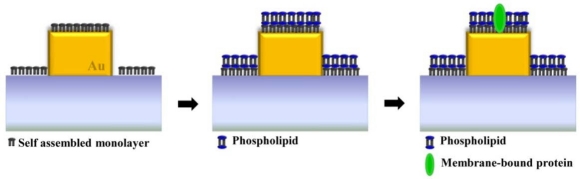
Detection of supported lipid membrane and membrane-bound protein at the gold nanostructure through LSPR peak shift.

A supported lipid bilayer (SLB) is an *in vitro* system that mimics the plasma membrane in living cells. SLB membranes can be made with a wide range of lipid compositions. They have sustained lateral mobility that bears resemblance to a natural cell membrane. Label-free kinetic measurements of biological membranes can be performed with a supported lipid bilayer. Real-time observations of the formation and mobility of SLB membranes have been explored by SPR spectroscopy instead of fluorescence tracking techniques [52]. Figure 13 illustrates lipid bilayers that are supported by hydrophilic self-assembled monolayer (SAM) onto the gold nanostructure. SAM is necessary to mount lipid membrane onto a noble metallic nanostructure. The difference in refractive index between a self assembled monolayer and a lipid membrane can be optically detected with the noble metal nanostructure. Membrane-bound proteins, which have a need of a lipid bilayer for intrinsic function, can be monitored via LSPR sensing as seen in Figure 13. Membrane-mediated cytotoxicity of a specific type of protein aggregates has attracted much interest due to its relation with conformational diseases. Specifically, neurodegenerative diseases, including Alzheimer’s disease, Parkinson’s disease, amyotrophic lateral sclerosis, Huntington’s disease, and Creutzfeldt–Jakob disease are caused by the extracelluar and intracellular deposition of misfolded proteins in the form of aggregates of insoluble amyloid [53]. Investigating the behavior of protein aggregates on a mimicking cell membrane is regarded as a valuable approach to gain knowledge about cellular component-mediated cytotoxic actions generated *in vivo*. These proteins act as the interface between a cell and its surroundings, interposing responses to growth factors and immune cells.

**Figure 14.**
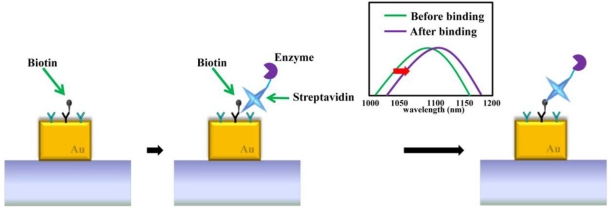
Detection of protein-protein binding event on the gold nanostructure through LSPR peak shift.

Furthermore, protein binding events are specifically detected via LSPR nano-sensors [54,55]. One of the typical proteins used for evaluating the performance of LSPR nano-sensors is biotin due to its strong affinity with other conjugate proteins [2,37]. Biotin is defined as a water soluble B-complex vitamin and is necessary for cell growth. Biotin strongly binds to avidin, streptavidin, and neutravidin as biological receptor/ligand pairs. Before detecting specific protein binding events, bovine serum albumin (BSA) is pre-used to eliminate non-specific binding interactions. Biotinylated bovine serum albumin was absorbed onto the gold nanostructures. The glass surface was subsequently blocked for protein binding by absorbing BSA before adding streptavidin solution. Figure 14 describes the sensing of a binding event between biotin and streptavidin on a gold nanostructure. After streptavidin binds with biotin, the resonant wavelength of the gold nanostructure is red-shifted. In an attempt to detect wavelength shifts caused by protein binding more accurately, the LSPR nano-sensor should be distinguished with the high spectral shift and narrow linewidth. In other examples of protein sensing, attomole levels of the protein myoglobin on a SERS substrate were detected on a gold nanograin aggregate array with interspacial distances down to 10 nm [12]. A highly reflective Al substrate interacting with nanoparticles was able to detect refractive index changes of the specific interaction of biomolecules including biotin and avidin, 5-fluorouracil and its antibody [56].

DNA can be detected by the aforementioned sensing method, single molecule detection. It is beneficial to detect a single molecule employing a few nanoscale structures such as nanodumbells [57]. These authors report SERS-active gold-silver core-shell nanodumbbells where the gap between two nanoparticles, Raman-dye position and environment can be engineered on the nanoscale. Single-DNA-tethered nanodumbells can be utilized to detect Raman signals. Besides, a new sensing modality to detect the target in a complex medium was proposed. Binding of target DNA takes to geometrical extension of nanoparticle dimers [58]. It yields a spectral blue shift in the hybridized plasmon mode. In addition, single-molecule detection of interleukin-2 by a silica microtoroid optical resonantor was demonstrated. Single-molecule binding event leads to the shift of the resonant frequency [59].

## 5. Conclusions

In this paper, we have reviewed notable nanostructures for plasmonic sensing and explained relative physical models. Optimized nanostructures exhibit not only intense field enhancement, but also high spectral resolution. It is claimed that specific biological detection can be associated with definite nanostructures. Table 1 presents the sorted characteristics of LSPR-based nanostructures. Diverse nanostructures have been exploited for achieving optimal LSPR nanosensors. Appropriate manipulation of geometrical parameters is a requirement for specific sensing purposes. However, certain design limitations prevent further research for complete geometry of LSPR sensor. Remaining challenges should be untangled, especially those concerning fabrication techniques and instrumental resolution.

## Figures and Tables

**Table 1.** Sensing characteristics of plasmonic nanostructures.

**Wavelength**	**Nanostructure**	**Structure Dimensions**	**Characteristic**	**Sensitivity**	**RI range**	**Ref.**
450–700 nm	Silver spherical nanoparticles	Diameter: 35 nm	Shape-dependent LSPR	161 nm/RIU	1–1.6	[18]
450–700 nm	Silver triangular nanoparticles	Diameter: 35 nm	Shape-dependent LSPR	197 nm/RIU	1–1.6	[18]
450–700 nm	Silver rodlike nanoparticles	Aspect ratio 5:1 Diameter: 35nm	Shape-dependent LSPR	235 nm/RIU	1–1.6	[18]
800–1,000 nm	Gold nano rods	Aspect ratio 3.5:1 Radius: 40 nm	Size-dependent LSPR	650 nm/RIU	1.34–1.7	[60]
450–600 nm	Gold colloidal nanoparticles	Diameter: 30 nm	Shape-dependent LSPR	70.9 nm/RIU	1.32–1.5	[61]
400–800 nm	Hollow gold nano shell	Diameter: 50 nm Wall thickness: 4.5 nm	Shape-dependent LSPR	408 nm/RIU	1.32–1.5	[61]
500–1,000 nm	Arrays of gold nanodisk	Pitch of nanodisk: 162 nm, 340 nm	Anisotropic property of gold nanodisk	167 nm/RIU 327 nm/RIU	1.32–1.42	[29]
600–750 nm 1,100–1,400 nm	Gold nanodisk trimers	Small disk diameter: 96 nm Larger disk diameter: 127 nm Pitch between trimers: 400 nm	Visible and near-infrared localized surface plasmon resonances	170 nm/RIU 373.9nm/RIU	1–1.5	[26]
900–1,500 nm	Gold nanorings	Diameter: 150 nm Thickness: 20 nm	Shape dependence on dielectric substrate	880 nm/RIU	1.33–1.42	[2]
500–1,700 nm	Gold nanoring trimers	Outer diameter:120 nm Wall thickness: 33 nm Ring height: 24 nm	Plasmon hybridization	345 nm/RIU	1–1.5	[27]
350–650 nm	Nanocubes	Size : 100 nm	Sharp quadripolar LSPR peak	165 nm/RIU	Neutravidin binding	[4]
800–2,800 nm	Nanocrescents	Diameter: 410 nm Deposition angle:10° Aspect ratio: 4	Plasmon-induced electromagnetic near-field	879 nm/RIU	Streptavidin binding	[3]
550–750 nm	Nanostars	Core size: 30–50 nm Conical tips: 10–60 nm	Multiple plasmon Resonances	218 nm/RIU	1–1.38	[37]
1200–1,900 nm	Nanocross and nanobar	Length: 380nm Width: 76 nm α = 60°	Subradiant Fano resonances	710 nm/RIU 1,000 nm/RIU	1.333–1.38	[38]
1,361 nm 1,512 nm	Double nanopillars with nanogap	Diameter: 425 nm Height: 288 nm Nanogap: 33 nm	Plasmon electromagnetic field induced by nanogap	642 nm/RIU 1,056 nm/RIU	1.34–1.44	[31]
950–2,500 nm	Planar metamaterials analogue of EIT	Length: 400 nm, 340 nm Width: 80 nm, 90 nm Gap: 45 nm	Electromagnetically induced transparency	725 nm/RIU	1.332–1.372	[35]
400–1,000 nm	Plasmonic nanorod metamaterials	Length: 20–700 nm Diameter: 10–50 nm Pitch: 40–70 nm	Anisotropic guided mode	30,000 nm/RIU	1–1.36	[41]
550–900 nm	Arrays of plasmonic nanotubes	Length: 120 nm Inner diameter: 22 nm Outer diamete: 44 nm Pitch: 55 nm	Shape-dependent LSPR	250 nm/RIU	1.333–1.368	[40]
750–1,000 nm	Nanopillar arrays	Radius: 100 nm Height: 400 nm Pitch: 600 nm	Monopole antenna arrays	675 nm/RIU	1.33–1.45	[39]

RI : Refractive Index

## References

[1] Tan Y.N., Su X., Zhu Y., Lee J.Y. (2010). Sensing of transcription factor through controlled-assembly of metal nanoparticles modified with segmented DNA elements. ACS Nano.

[2] Larsson E.M., Alegret J., Kall M., Sutherland D.S. (2007). Sensing characteristics of NIR localized surface plasmon resonances in gold nanorings for application as ultrasensitive biosensors. Nano Lett.

[3] Bukasov R., Ali T.A., Nordlander P., Shumaker-Parry J.S. (2010). Probing the plasmonic near-field of gold nanocrescent antennas. ACS Nano.

[4] Galush W.J., Shelby S.A., Mulvihill M.J., Tao A., Yang P., Groves J.T. (2009). A nanocube plasmonic sensor for molecular binding on membrane surfaces. Nano Lett.

[5] Kim D.K., Park T.J., Tamiya E., Lee S.Y. (2011). Label-free detection of leptin antibody-antigen interaction by using LSPR -based optical biosensor. J. Nanosci. Nanotechnol.

[6] Stewart M.E., Anderton C.R., Thompson L.B., Maria J., Gray S.K., Rogers J.A., Nuzzo R.G. (2008). Nanostructured plasmonic sensors. Chem. Rev.

[7] Lee B., Roh S., Park J. (2009). Current status of micro-and nano-structured optical fiber sensors. Opt. Fiber Technol.

[8] Svedendahl M., Chen S., Dmitriev A., Kall M. (2009). Refractometric sensing using propagating versus localized surface plasmons. Nano Lett.

[9] Anker J.N., Hall W.P., Lyandres O., Shah N.C., Zhao J., Van Duyne R.P. (2008). Biosensing with plasmonic nanosensors. Nat. Mater.

[10] Jain P.K., El-Sayed M.A. (2010). Plasmonic coupling in noble metal nanostructures. Chem. Phys. Lett.

[11] Oh Y.-J., Park S.-G., Kang M.-H., Choi J.-H., Nam Y., Jeong K.-H. (2011). Beyond the SERS: Raman enhancement of small molecules using nanofluidic channels with localized surface plasmon resonance. Small.

[12] Das G., Mecarini F., Angelis F.D., Prasciolu M., Liberale C., Patrini M., Fabrizio E.D. (2008). Attomole myoglobin Raman detection from plasmonic nanostructures. Microelectron. Eng.

[13] Homola J. (2006). Electromagnetic Theory of Surface Plasmons. Surface Plasmon Resonance Based Sensors.

[14] Liedberg B., Nylander C., Lunstrom I. (1983). Surface plasmon resonance for gas detection and biosensing. Sens. Actuat.

[15] Liao P.F., Wokaun A. (1982). Lightning rod effect in surface enhanced Raman scattering. J. Chem. Phys.

[16] Zhu J., Li F.-K. (2011). Effect of aspect ratio on the inter-surface plasmonic coupling of tubular gold nanoparticle. Eur. Phys. J. B.

[17] Jain P.K., El-Sayed M.A. (2008). Noble metal nanoparticle pairs: Effect of medium for enhanced nanosensing. Nano Lett.

[18] McFarland A.D., Van Duyne R.P. (2003). Single silver nanoparicles as real-time optical sensors with zeptomole sensitivity. Nano Lett.

[19] Kim H.M., Jin S.M., Lee S.K., Kim M., Shin Y. (2009). Detection of biomolecular binding through enhancement of localized surface plasmon resonance (LSPR) by gold nanoparticles. Sensors.

[20] Wiley B.J., Chen Y., McLellan J.M., Xiong Y., Li Z.-Y., Ginger D., Xia Y. (2007). Synthesis and optical properties of silver nanobars and nanorice. Nano Lett.

[21] Wei H., Reyes-Coronado A., Nordlander P., Aizpurua J., Xu H. (2010). Multipolar plasmon resonances in individual Ag Nanorice. ACS Nano.

[22] Hanarp P., Kall M., Sutherland D.S. (2003). Optical properties of short range ordered arrays of nanometer gold disks prepared by colloidal lithography. J. Phys. Chem. B.

[23] Wu L.Y., Ross B.M., Lee L.P. (2009). Optical properties of the crescent-shaped nanohole antenna. Nano Lett.

[24] Prodan E., Radloff C., Halas N.J., Nordlander P. (2003). A hybridization model for the plasmon response of complex nanostructures. Science.

[25] Lassiter J.B., Azipurua J., Hernandez L.I., Brandl D.W., Romero I., Lai S., Hafner J.H., Nordlander P., Halas N.J. (2008). Close encounters between two nanoshells. Nano Lett.

[26] Tripathy S., Mlayah A. (2010). Dual wavelength sensing based on interacting gold nanodisk trimers. Nanotechnology.

[27] Lin V.K., Teo S.L., Marty R., Arbouet A., Girard C., Alarcon-Llado E., Liu S.H., Han M.Y., Teo S.L., Lin V.K. (2010). Gold nanoring trimers: A versatile structure for infrared sensing. Opt. Express.

[28] Hentsch M., Dregely D., Vogelgesang R., Giessen H., Liu N. (2011). Plasmonic oligomers: The role of individual particles in collective behavior. ACS Nano.

[29] Lee S.-W., Lee K.-S., Ahn J., Lee J.-J., Kim M.-G., Shin Y.-B. (2011). Highly sensitive biosensing using arrays of plasmonic Au nanodisks realized by nanoimprint lithography. ACS Nano.

[30] Hao F., Nehl C.L., Hafner J.H., Nordlander P. (2007). Plasmon resonances of a gold nanostar. Nano Lett.

[31] Kubo W., Fujikawa S. (2011). Au double nanopillars with nanogap for plasmonic sensor. Nano Lett.

[32] Hrelescu C., Sau T.K., Rogach A.L, Jackel F., Laurent G., Douillard L., Charra F. (2011). Selective excitation of individual plasmonic hotspots at the tips of single gold nanostars. Nano Lett.

[33] Zhang S., Bao K., Halas N.J., Xu H., Nordlander P. (2011). Substrate-induced Fano resonances of a plasmonic nanocube: A route to increased-sensitivity localized surface plasmon resonance sensors revealed. Nano Lett.

[34] Gomez D.E., Vernon K.C., Davis T.J. (2010). Symmetry effects on the optical coupling between plasmonic nanoparticles with applications to hierarchical structures. Phys. Rev. B.

[35] Liu N., Weiss T., Mesch M., Langguth L., Eigenthaler U., Hirscher M., Sonnichsen C., Giessen H. (2010). Planar metamaterial analogue of electromagnetically induced transparency for plasmonic sensing. Nano Lett.

[36] Vogel N., Fischer J., Mohammadi R., Retsch M., Butt H.-J., Landfester K., Weiss C.K., Kreiter M. (2011). Plasmon hybridization in stacked double crescents arrays fabricated by colloidal lithography. Nano Lett.

[37] Dondapati S.K., Sau T.K., Hrelescu C., Klar T.A., Stefani F.D., Feldmann J. (2010). Label-free biosensing based on single gold nanostars as plasmonic transducers. ACS Nano.

[38] Verellen N., Dorpe P.V., Huang C., Lodewijks K., Vandenbosch G.A.E., Lagae L., Moshchalkov V.V. (2011). Plasmon line shaping using nanocrosses for high sensitivity localized surface Plasmon resonance sensing. Nano Lett.

[39] Cetin A.E., Yanik A.A., Yilmaz C., Somu S., Busnaina A., Altug H. (2011). Monopole antenna arrays for optical trapping, spectroscopy, and sensing. Appl. Phys. Lett.

[40] McPhillips J., Murphy A., Jonsson M.P., Hendren W.R., Atkinson R., Hook F., Zayats A.V., Pollard R.J. (2010). High-performance biosensing using arrays of plasmonic nanotubes. ACS Nano.

[41] Kabashin A.V., Evans P., Pastkovsky S., Hendren W., Wurtz G.A., Atkinson R., Pollard R., Podolskiy V.A., Zayats A.V. (2009). Plasmonic nanorod metamaterials for biosensing. Nat. Mater.

[42] Greffet J.-J. (2005). Nanoantennas for light emission. Science.

[43] Park Q.-H. (2009). Optical antennas and plasmonics. Contemp. Phys.

[44] Pakizeh T., Kall M. (2009). Unidirectional ultracompact optical nanoantennas. Nano Lett.

[45] Yu N., Cubukcu E., Diehl L., Bour D., Corzine S., Zhu J., Hofler G., Crozier K.B, Capasso F. (2007). Bowtie plasmonic quantum cascade laser antenna. Opt. Express.

[46] Kinkhabwala A., Zongfu Y., Fan S., Avlasevich Y., Mullen K., Moerner W.E. (2009). Large single-molecule fluorescence enhancements produced by a bowtie nanoantenna. Nat. Photonics.

[47] Aouani H., Mahboub O., Bonod N., Devaux E., Popov E., Rigneault H., Ebbesen T.W., Wenger J. (2011). Bright unidirectional fluorescence emission of molecules in a nanoaperture with plasmonic corrugations. Nano Lett.

[48] Kosako T., Kadoya Y., Hofmann H.F. (2010). Directional control of light by a nano-optical Yagi-Uda antenna. Nat. Photonics.

[49] Taminiau T.H., Stefani F.D., Van Hulst N.F. (2008). Enhanced directional excitation and emission of single emitters by a nano-optical Yagi-Uda antenna. Opt. Express.

[50] Wang Y., Qian W., Tan Y., Ding S. (2008). A label-free biosensor based on gold nanoshell monolayers for monitoring biomolecular interactions in diluted whole blood. Biosens. Bioelectron.

[51] Marinakos S., Chen S., Chilkoti A. (2007). Plasmonic detection of a model analyte in serum by a gold nanorod sensor. Anal.Chem.

[52] Maynard J.A., Lindquist N.C., Sutherland J.N., Lesuffleur A., Warrington A.E., Rodriguez M., Oh S.-H. (2009). Surface plasmon resonance for high-throught ligand screening of membrane-bound proteins. Biotechnol. J.

[53] Krazinski B.E., Radecki J., Radecka H. (2011). Surface plasmon resonance based biosensors for exploring the influence of alkaloids on aggregation of amyloid-β Peptide. Sensors.

[54] Raschke G., Kowarik S., Sonnichsen F.C., Klar T.A., Feldmann J., Nichtl A, Kurzinger K. (2003). Biomolecular recognition based on single gold nanoparticle light scattering. Nano Lett.

[55] Xu J., Zhang L., Gong H., Homola J., Yu Q. (2011). Tailoring plasmonic nanostructures for optimal SERS sensing of small molecules and large microorganisms. Small.

[56] Hiep H.M., Yoshikawa H., Tamiya E. (2010). Interference localized surface plasmon resonance nanosensor tailored for the detection of specific biomolecular interactions. Anal. Chem.

[57] Lim D.K., Jeon K.-S., Kim H.-M., Nam J.-M., Suh Y.D. (2010). Nanogap-engineerable Raman-active nanodumbbells for single-molecule detection. Nat. Mater.

[58] Chen J.I.L., Chen Y., Ginger D.S. (2010). Plasmonic nanoparitlce dimers for optical sensing of DNA in complex media. J. Am. Chem. Soc.

[59] Armani A.M., Kulkarni R.P., Fraser S.E., Flagan R.C., Vahala K.J. (2007). Label-free, single molecule detection with optical microcavities. Science.

[60] Lee K.-S., El-Sayed M.A. (2006). Gold and silver nanoparticles in sensing and imaging. J. Phys. Chem. B.

[61] Sun Y., Xia Y. (2002). Increased sensitivity of surface plasmon resonance of gold nanoshells compared to that of gold solid colloids in response to environmental changes. Anal. Chem.

